# Effects of multilevel metacognition on group performance and regulation in collaborative learning

**DOI:** 10.3389/fpsyg.2024.1419408

**Published:** 2024-11-29

**Authors:** Lifang Qiao, Wei Zhao, Fengjuan Liu, Xiaoqing Xu, Jinhong Tao

**Affiliations:** ^1^School of Information Science and Technology, Northeast Normal University, Changchun, China; ^2^Department of Education Science, Shaanxi University of Technology, Hanzhong, China

**Keywords:** multilevel metacognition, group metacognition, individual metacognition, interpersonal metacognition, collaborative learning

## Abstract

Group metacognition is essential for effective collaboration. However, existing research mainly focuses on individual metacognition in collaborative learning, and some studies focused on interpersonal metacognition. The understanding of group metacognition is limited. Less attention has been paid to how multilevel metacognition, including individual metacognition, interpersonal metacognition, and group metacognition, functions. There is also less research on whether group metacognition influences collaborative learning through other levels of metacognition. To investigate the emergence of multilevel metacognition among learners with varying achievement and its effects on group performance and regulation patterns, this study employed both traditional and process analyses to examine how the distribution and interactions of multilevel metacognition influence group performance and group regulation. The study revealed that in collaborative learning, group metacognition exerts the most significant influence on group performance compared to other metacognitive levels, such as individual metacognition and interpersonal metacognition. Specifically, the study identified three collaborative achievement categories by judging the degree of collaborative benefit based on individual preparation and group performance: H_T category, EF category and L_T category. The H_T and EF categories performed better and developed more group metacognition, while the L_T category exhibited poorer performance and less group metacognition. The study highlights the role of other levels of metacognition in forming group metacognition, with multilevel metacognitive interactions elucidating the mechanisms of group metacognition. These insights provide practical insights for multilevel metacognition and offers guidance for collaborative learning interventions, particularly those targeting group metacognition.

## 1 Introduction

Collaborative learning promotes active learner engagement through social interactions and fosters high-level awareness, better control of the learning process, and positive interdependence and responsibility connections among learners (Tang et al., 2022). However, achieving efficient collaboration requires high-quality participatory interactions among group members to manage the dynamic nature of groups and foster effective collaboration (Badhe et al., 2023). Metacognitive interactions during group processes play a pivotal role in effective collaboration (Bakhtiar and Hadwin, 2020). Metacognitive interaction is the process by which groups in collaborative learning monitor and regulate their learning processes, including setting goals, planning, monitoring, and evaluating (Molenaar et al., 2011). Metacognitive interaction involves multiple levels of metacognition: individual, interpersonal, and group. Individual Metacognition (IM) refers to the process by which individuals monitor and regulate their cognitive activities to improve their collaborative participation. Interpersonal metacognition (IPM) encompasses the sharing of metacognitive strategies among members, along with providing mutual support to facilitate group learning. Group metacognition (GM) entails the collective monitoring and regulation of cognitive processes within the group. Influenced by the social environment, metacognition during group interactions gradually evolves from the individual level to the interpersonal level and, ultimately, to the group level (Iiskala et al., 2021; Waller et al., 2021). There is a pressing need to explore metacognitive interaction from a multilevel perspective to understand how groups collaborate for learning success. However, existing studies have primarily explored metacognitive interactions by focusing on level-specific metacognition, such as IM development, the effectiveness of collaborative learning through IPM (Briñol and DeMarree, 2012) or the level and impact of GM (Biasutti and Frate, 2018). Research on level-specific metacognition has concentrated on IM, with little attention given to GM. However, research on metacognition in collaborative learning has shifted focus from the individual to the group, recognizing GM as a key component of successful collaboration (Rapchak, 2018). More researchers are advocating for increased exploration of GM (Biasutti and Frate, 2018; Liu et al., 2023). Moreover, studies have demonstrated positive effects of metacognition across different levels (Kim and Moore, 2019; Socratous and Ioannou, 2022). However, the interaction between different levels has not been comprehensively explored. Research on how to develop GM is still in the early stages. Researchers need to explore the role of individuals in groups by identifying different levels of metacognition and revealing how GM influences the process and performance of collaborative learning. Furthermore, metacognitive interaction in collaborative learning unfolds sequentially as a temporal process. Sequentiality refers to when behaviors occur and how they interact with each other over time. Sequential methods and stochastic views of sequences are well suited for conversational analysis, enabling the exploration of temporality and enhancing the understanding of group processes (Saqr and López-Pernas, 2023). Sequential analyses of metacognitive interaction processes, particularly how multilevel metacognition interacts, contribute to a deeper understanding of group regulation (Iiskala et al., 2021). However, only a few studies have focused on the temporal and sequential aspects of metacognitive interaction (Su et al., 2018; Zheng et al., 2023). There is a need for comprehensive exploration of how multilevel metacognition, IM, IPM, and GM, change over time during collaborative interactions, influencing group regulation and group performance. Group regulation refers to the patterns formed through the interactions of various levels of metacognition within the group, while group performance represents the collective learning outcomes resulting from these interactions. Therefore, based on statistical analysis, this study proposes a fusion sequence analysis approach (sequence analysis fused with sequence mining) to investigate the multilevel characteristics of metacognitive interactions. The objective is to determine how group learners form GM through IM and IPM to explain effective collaboration.

### 1.1 Cooccurrence of multilevel metacognition in the collaborative learning group

#### 1.1.1 Multilevel metacognition in group process collaborative learning

The level of participation and impact of metacognitive activities within a group depend on members’ subsequent metacognitive contributions after the initial trigger and their influence on the group in collaboration. This illustrates how members within a group, among themselves, and as a whole regulate their metacognition to achieve common goals, forming the basic process of metacognitive interaction. This highlights the multilevel nature of metacognitive interaction, blending individual tendencies and group influence (Kelly, 2018; Waller et al., 2021), involving individuals in the group and the group as social entities. Metacognition in collaborative learning can be divided into three levels based on the participants and actors involved in metacognitive interaction: IM, IPM, and GM (Thompson and Cohen, 2012; Iiskala et al., 2021).

IM focuses on how individuals monitor and control their cognitive processes, such as task understanding, planning, monitoring, evaluation, and reflection (Meijer et al., 2006). In metacognitive interactions, learners externalize IM by sharing their thoughts and selection strategies for collaborative tasks. The participants and influencers in this process were all individual learners. Research has shown that more successful individuals exhibit more metacognitive processes in collaborative learning, and learners with higher levels of metacognition may guide metacognitive interaction (Smith and Mancy, 2018). While the role and developmental changes in IM are the main focus of relevant research, it is also important to explore how individuals impact groups and promote collaboration.

IPM, dominated by individuals, focuses on how individuals monitor and control the cognition of group members (Molenaar et al., 2014) without being constrained by group goals (Tang et al., 2022). The participants were individuals within the group, while the influencers were group members. This concept emphasizes the awareness and regulation of others’ thinking for learning purposes (Stanton et al., 2021), which can help individuals better understand and adapt to social interaction environments. By stimulating each other’s metacognitive processes (Halmo et al., 2022), effective collaboration among learners can be promoted. Studies have demonstrated the interconnectedness of the IM and IPM (Haataja et al., 2022a,b; Kelly, 2018; Smith and Mancy, 2018). However, effective collaboration within a group is contingent upon these interpersonal influences being aligned with common goals. Without such alignment, the impact on group-level collaboration might be minimal (Hurme et al., 2015).

GM views a group as a whole, focusing on how the group monitors and regulates collective cognition to ensure its accuracy and enhance group decision-making ability. Both the participants and influencers are the group. GM can be seen as an extension of learner metacognition in interpersonal interactions (Socratous and Ioannou, 2019). The early concept of GM was similar to social metacognition, emphasizing group members’ cognition and regulation. Currently, GM stresses its cognitive and regulatory role in group cognition following metacognitive interactions focused on individuals (Siegel, 2012). When interpersonal shared cognitions are considered group primary thoughts, learners can recognize and regulate these shared thoughts at the group level (Briñol and DeMarree, 2012). GM ensures the achievement of collaborative goals and improves the quality of group decision-making and the efficiency of group tasks.

#### 1.1.2 Cooccurrence of multilevel metacognition

IM, IPM, and GM coexist in interactions (Hurme et al., 2015; Molenaar et al., 2014). Studies increasingly emphasize the importance of the GM. For instance, Haataja et al. reported a positive correlation between metacognitive interaction and group performance, highlighting the significance of metacognitive interaction and individual monitoring in relation to achievement (Haataja et al., 2022a). Additionally, Huang et al. found that IM has a limited impact on group performance in online collaborative learning, emphasizing the need for more attention to GM (Huang et al., 2021). Iiskala et al. (2011) revealed the interconnection between the IM and the GM, a relationship that deserves further investigation. Molenaar et al. explored the relationship between different types of social metacognitive interactions and learners’ metacognitive knowledge, emphasizing the importance of distinguishing levels of metacognitive interaction in groups (Molenaar et al., 2014). Furthermore, exploring how the IM can be extended to the GM is necessary (Huang et al., 2021; Iiskala et al., 2011; Socratous and Ioannou, 2022). A comprehensive examination of how different levels of metacognition interact to support the development of the GM is necessary (Halmo et al., 2022). Moreover, understanding the metacognitive interaction of complex groups through technology deserves attention (Järvelä et al., 2021). Empirical research on the relationship between GM and learners’ performance is still limited (Haataja et al., 2022a) but may offer insights into how metacognitive interaction affects group performance.

### 1.2 The role of group metacognition in collaborative learning

Emphasizing GM in collaborative learning allows researchers to focus on interactions between members and purposeful group coordination (Kwon et al., 2013). Specifically, a group member’s metacognition contributes to discussing how to handle group tasks, activating the group metacognitive process. When other members recognize the similarities of their metacognition and further develop this information, GM begins to develop. Subsequently, when this information impacts a group’s direction or outcome, the GM plays a pivotal role (Hurme et al., 2009). In collaboration, the GM promotes the continuation of proper cognitive processes or prevents collective cognition from developing in the opposite direction (Iiskala et al., 2011). Through GM, group members can adjust their collective cognition based on their common goals, addressing lower levels of perceptual difficulties (Hurme et al., 2009).

The role of GM in collaborative learning is evident in various ways. First, its critical importance has been demonstrated, and there is a focus on measuring its impact on learning and behavior. Chalmers define GM as including group planning, monitoring, and evaluation (Chalmers, 2009). Biasutti et al. proposed that GM can be assessed through four dimensions: group cognitive knowledge, planning, monitoring, and evaluation (Biasutti and Frate, 2018). Siegel et al. suggested that GM comprises meta-social awareness, monitoring understanding, and monitoring processes, which develop through acceptance, rejection, and recombination (Siegel, 2012). Zheng et al. described group metacognitive behavior as orientation, planning, executing strategies, monitoring and control, evaluation and reflection, and adaptive metacognition (Zheng et al., 2019). Considering the impact of goals on metacognitive interaction and the need to distinguish among different levels of metacognition, this study conceptualizes GM across four dimensions: group orientation, group planning, group monitoring, and group evaluation. Second, the focus has shifted to enhancing GM to promote collaboration. For instance, Liu et al. reported that social sharing regulation supports the improvement of learners’ GM (Liu et al., 2023). Li et al. discovered that collaboration based on metacognitive regulation is beneficial for enhancing learners’ GM (Li et al., 2023). Socratous and Ioannou (2022) observed that learners in structured courses exhibit higher levels of GM and collaborative learning performance than those in unstructured courses. Structured course group provided direct instruction, significantly increasing GM and improving collaboration quality. In contrast, unstructured course group, although offering more autonomy, did not effectively support learners in developing GM through productive failure. Finally, some studies have investigated how GM improve collaborative learning (Teng, 2020; Uslu and Durak, 2022; Zheng et al., 2019). For example, Teng (2020) explored the effects of GM support on metacognitive awareness and learning performance. Zheng et al. (2019) investigated the impact of group metacognitive scaffolding on group metacognitive behavior and performance. Other studies have examined the influence of GM on learners (Uslu and Durak, 2022).

Existing studies have highlighted the significant role of GM in collaborative learning, primarily focusing on its impact on collaborative learning performance. However, there is limited research on how successful groups engage in GM from a process perspective, particularly in understanding group patterns. Longitudinal research on how the GM changes over time deserves attention (Zheng et al., 2019) to provide targeted support for the GM. Additionally, while some studies examine the group’s metacognitive process from a regulatory perspective, there is a lack of analysis distinguishing levels of metacognitive interactions based on the participants and influencers. For instance, the GM has been explored as a complex shared learning dynamic or coregulation (Olesova et al., 2023). Further research is necessary to comprehend how learners progress toward GM in collaboration (Olesova et al., 2023). Moreover, existing studies have focused predominantly on how traditional statistical methods measure the GM, with some researchers using the chronologically ordered representation diagram for tool-related activity (CORDTRA) to offer initial evidence on how the GM in collaboration changes over time (Socratous and Ioannou, 2019). Therefore, reliable methods capable of analyzing GM in large-scale collaboration datasets are essential for bridging this gap (Liu et al., 2023; Tang et al., 2022).

The purpose of this study is to investigate how multilevel metacognition affects collaborative learning. The study focuses on the effects of different levels of metacognition on group performance, as well as the effects of sequential interactions of other levels of metacognition on group regulation.

Specifically, the study identifies three achievement group categories, revealing the specific effects of each category on group performance. By analyzing the distribution and differences of multilevel metacognition across these categories, the study aims to elucidate how multilevel metacognition influences group regulation. The following questions will be addressed.

This study aimed to investigate how multilevel metacognition influence collaborative learning focusing on the effects of multilevel metacognition on group performance, as well as the effects of sequential interactions of multilevel metacognition on group regulation. Specifically, the study identifies three achievement group categories, revealing the specific effects of each category on group performance. By analyzing the distribution and differences of multilevel metacognition across these categories, the study aims to elucidate how multilevel metacognition influences group regulation. The following questions will be addressed:

RQ1: What is the distribution of multilevel metacognition (IM, IPM and GM), across group categories with varying achievements in collaborative learning, and how it affect group performance?

RQ2: What are the sequential characteristics and patterns of multilevel metacognition across learner groups with different achievements, and how do interactions of multilevel metacognition influence group regulation?

## 2 Materials and methods

### 2.1 Participants and setting

This study examines online collaborative learning in higher education in China, specifically within the undergraduate course titled “Teaching Media and Technology,” which was offered during the spring semester of 2022. Collaborative learning tasks are the main learning activities and are designed to facilitate group-based design and practical problem solving related to teaching media and technology. Research consent forms were distributed through social media (WeChat) to invite students to participate, resulting in 66 participants. Drawing on prior knowledge, researchers formed 14 heterogeneous groups, each consisting of at least four participants. However, one group withdrew midway due to members’ inability to engage in real-time collaborative tasks. Therefore, 13 groups with a total of 61 participants were included in the analysis.

### 2.2 Collaborative learning environment and tasks

The online collaborative learning environment is facilitated by Tencent Meeting, which provides features such as text chat, audio and video communication, screen sharing, notes and comments, resource sharing, and collaborative editing. During collaboration, group members initially review task requirements through an online video conference, share their understanding and opinions, confirm goals and tasks, and then allocate tasks. Subsequently, the group gathers collectively shared resources and finalize the group plan. Members then communicate offline to support individual task completion through voice and text chat and upload completed personal tasks. Finally, during the second video conference, members share the status of completion, evaluate and provide suggestions, monitor task progress, negotiate modifications, and ultimately complete the group task. Learners’ collaborative task performance was assessed based on two main components: (1) individual preparation performance (40%), evaluating the completion of the individual task; and (2) group task performance (60%), assessing the completion and quality of the group’s collaborative task. This collaborative task contributes to 20% of the overall course performance.

### 2.3 Data collection

In this study, computer screen videos were collected to capture data during collaborative learning, with each group producing two video conference records for every task. Groups are more likely to employ GM in more complex learning tasks. Therefore, the fourth collaborative learning task, which involved designing a teaching model using teaching media and technology, was selected for exploring GM. All the groups had no prior experience with the task and possessed similar levels of prior knowledge. Furthermore, following the completion of three collaborative learning tasks, group members became more familiar with each other, improved their utilization of online collaboration platforms, and developed a deeper understanding of effective collaboration. Ultimately, 26 segments, totaling about 20 h video data, were collected (avg: 1.5 h; min: 0.8 h; max: 2.38 h).

#### 2.3.1 Data coding

Based on the literature, we encoded the group metacognitive process (Table 1). It is crucial for researchers to examine the influence of cognition and social experience on learners’ engagement in metacognitive interactions (De Backer et al., 2022) and to characterize the entire collaborative process. We encoded cognition and social behavior to gain a more precise understanding of the group’s metacognitive interaction. The research divides metacognitive interaction into independent segments based on the end of the discussion or interrupted discourse and then encodes the events within each segment. In this study, GM events were defined as the stage at which participants completed collaborative learning tasks, during which participants collectively regulated group cognition based on the group goal. To identify GM, the study conducted the following three preparatory procedures in advance.

**Table 1:** Metacognitive interaction coding framework.

Levels	Events	Description	Example
IM	Orientation (IM_O)	Discuss personal goals and task understanding, activate individual prior knowledge	“My goal is to improve my instructional design skills and actively participate in group discussions.”
	Plan (IM_P)	Develop a plan for personal task scheduling, time management, and strategy selection	“I plan to complete the portion of the assignment I’m responsible for by next Wednesday.”
	Monitoring (IM_M)	Monitor individual task understanding, task completion, and progress	“I have realized that the second part of my assignment appears to lack a clear connection to the task assigned to B.”
	Evaluation (IM_E)	Evaluate individual learning content, process, plan, and achievements	“While I achieved my goals, I realized that my intense focus on expressing my views occasionally caused me to lose sight of the group’s overall process.”
IPM	Orientation (IPM_O)	Discuss understanding of group members’ learning goals and tasks, acquire knowledge and beliefs of group members, and activate their prior knowledge	“You need to strengthen your focus on collaborative learning skills.”
	Plan (PIM_P)	Assist group members in developing a plan for task scheduling, time management, and strategy selection	“You could complete the case study before beginning the individual task.”
	Monitoring (IPM_M)	Monitor the task understanding, completion status, and progress of group members	“Is your second part of the assignment in line with the requirements of the task?”
	Evaluation (IPM_E)	Evaluate the learning content, process, plan, and achievements of group members	“You completed the task very well!”
GM	Orientation (GM_O)	Discuss group goals and task understanding, acquire knowledge and beliefs of group members, and activate group prior knowledge	“Our group should learn how to be more efficient while acquiring knowledge”
	Plan (GM_P)	Develop a plan for group task scheduling, time management, and strategy selection	“We plan to hold the second phase of discussions next Friday to confirm the progress of the modifications and enhance the group’s tasks.”
	Monitoring (GM_M)	Monitor group direction, task understanding, task completion, progress, and collaboration	“We have basically completed the first stage of discussion and identified the existing problems.”
	Evaluation (GM_E)	Evaluate group learning content, process, plan, achievements, and collaboration.	“The group goals have been achieved, and our division of labor is well-organized, but there is a phenomenon of procrastination.”
Cognition (C)		Share task information, personal task content, repeat task-related content, describe task processing, explain task content and extrapolate or summarize task content	“The ASSURE model comprises six segments.”
Others (Ot)		Perform social coordination, off-task statements and other unclassifiable behaviors	“Do not worry, we can talk about it together.”

First, it is essential to distinguish between cognitive codes and metacognitive codes. This study follows Nelsons (1999) ‘relative difference’ criterion, defining ‘metacognitive activities as activities that use information from cognitive activities or control cognitive activities through modification’. For example, “sharing task completion” is encoded as cognitive behavior, while “evaluating task completion” is classified as metacognitive behavior. Social behaviors are coded as other behaviors.

Second, episodes are identified by recognizing the interactive turns in the video. In interactive situations, a turn is typically defined as a continuous unit of communication that starts and ends with one person and transitions with a response from another. For instance, if Person A begins to explain, Person A’s turn starts. If Person B interrupts, this signifies the end of Person A’s turn and the beginning of Person B’s turn. Subsequently, if Person C responds to Person B, it indicates that Person B’s turn has ended and Person C’s turn has started. In the video interaction, a turn occurs when a member’s discourse or actions are interrupted by peers’ discourse or actions (Iiskala et al., 2011). When coding the utterance in a turn, attention was paid to whether the meaning of the utterance was divisible. If it was divisible, it was coded according to its meanings. If it was not divisible, it was coded according to the predominant purpose of its meaning. Typically, a group metacognitive segment comprises three levels of metacognition, two key discourses, and one group consensus. The three levels include the IM, IPM, and GM, which are formed by the support of the first two levels. The two discourses refer to the initiation and suspension or termination of collaborative dialog (Iiskala et al., 2011). A group consensus refers to the participation of more than half of the members in the discussion, leading to a consistent group understanding after the collaborative dialog.

Finally, we focused on the three levels and four events in metacognitive interaction. The three levels refer to individual, interpersonal, and group metacognition (see Table 1). This study primarily discerns the levels of metacognition through participants and influencers in metacognitive interaction. IM involves metacognitive dialog directed toward self-questioning or self-explanation, influencing only the individual learner. ‘I’ is usually used to indicate the subject’s intention. IPM refers to interactions guided by mutual assistance in completing individual tasks involving a guiding or reciprocal relationship between members. Usually, ‘you’, or member names, are used to identify the affected individuals. GM refers to interactions guided by shared goals to form GM and accomplish group tasks. The use of ‘we’ emphasizes group decision-making. The four events include goal setting, planning, monitoring, and evaluation (see Table 1). Two researchers coded the video data (200 turns) based on metacognitive interaction coding framework. Cohen’s kappa value was 0.91 (95%CI: 0.85–0.97, *p* < 0.001), indicating good agreement (Fleiss, 1981).

#### 2.3.2 Analytical methods

To effectively differentiate learner differences, clusters were delineated using latent profile analysis (LPA). The LPA explains the correlation between explicit indicators by determining potential categories. The optimal number of profiles was determined by the Akaike information criterion (AIC) and Bayesian information criterion (BIC), with smaller values indicating better model selection results. This study used the R package mclust to conduct LPA and determine group categories.

For the distribution of different levels of metacognition within a group of learners and to what extent GM is established (RQ1), this study used descriptive statistics to understand the distribution of different levels of metacognition within a category. One-way analysis of variance (ANCOVA) was also used to assess the impact of GM on students’ collaborative learning performance.

To characterize and identify the sequential features and patterns of metacognitive interaction among learners with different achievements (RQ2), stochastic process analysis was used. Considering the impact of single-method flatness on result accuracy, this study integrates sequence analysis using Lag Sequential Analysis (LSA) via the lagseq package in R (version 4.3.1) to identify key sequence features and sequence mining implementing cSPADE via the arulesSequences package in R (version 4.3.1) to investigate sequence rules and patterns among different categories, complementing the LSA results. LSA is used to analyze the lag effect in behaviors or time series, focusing on the temporal correlation between pairs of events and identifying behavioral characteristics. In contrast, sequence mining reveals relationships between consecutive events, focusing on discovering meaningful patterns to identify frequent rules and patterns in ordered events. Direct and indirect sequential meanings complement each other to support a deeper understanding of sequential dynamics. Specifically, LSA calculates transfer probabilities or frequencies between events by constructing transfer matrices between target events and further tests whether the transitions are significant using *z*-scores, which indicate significant transitions when *z* > 1.96. Sequence mining uses algorithms to discover frequent rules or patterns based on support and confidence. Support measures how often a sequence in a frequent rule occurs in the entire set of sequences, while confidence indicates the probability of occurrence of a sub-sequence given that the frequent rule contains a preceding sequence.

## 3 Results

**Figure 1:**
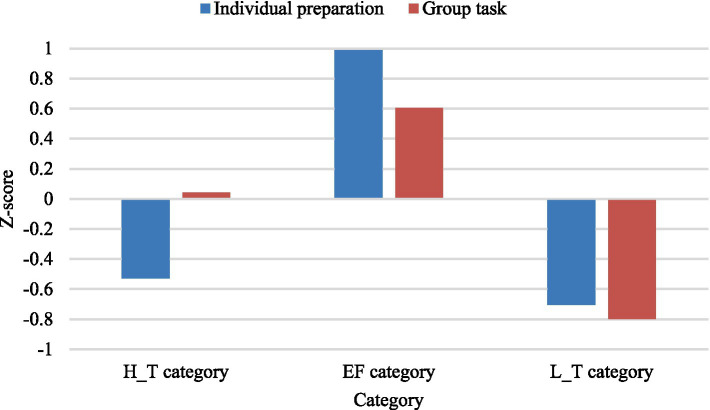
Category recognition based on group performance.

This study used LPA to categorize the initial groups based on individual preparations and group task performance, resulting in distinct achievement levels. Figure 1 indicates that when the number of categories is 3, the values for AIC (262), BIC (276), and entropy (0.989) are minimized, suggesting a reasonable division. The collaborative learning performance of the different categories is shown in Table 2. Category 1, comprising 20 learners, included four groups. This achievement category exhibited a low score for individual preparation, but the most significant improvement in the group task was achieved through collaboration. Thus, this group was called the high-transactivity collaborative achievement category (H_T category). In Category 2, the scores for both individual preparation and group tasks are higher, signifying effective collaboration among learners in these groups; hence, this group is referred to as the effective collaborative achievement category (EF category) and contains five groups, with 23 learners. Category 3, named the low-transactivity collaborative achievement category (L_T category), had four groups with 19 participants and demonstrated lower scores for individual preparation and group tasks, suggesting ineffective collaboration compared to other learner categories. The Kruskal–Wallis test revealed a significant difference in individual preparation performance between the categories (*p* = 0.016 < 0.05). However, there was no significant difference in group task performance between the categories (*p* = 0.131 > 0.05). Post-hoc test using Dunn’s test indicated a significant difference between the EF category and L_T category regarding group task performance. These results suggest that different categories performed differently in the individual preparation, and some of these differences were partially mitigated through group work. This reflects the fact that different categories benefited differently from collaboration. Although overall differences in group task performance were not significant, significant differences existed between particular categories, revealing intra-category heterogeneity. It is essential to understand the dynamics of collaboration within different categories to explain the extent of benefits and the reasons for these differences.

**Table 2:** Group performance of different categories.

Category	*N*	Individual preparation performance	Group task performance
H_T category	20	23.9	33.6
EF category	23	26.1	34.8
L_T category	19	24.2	31.8

*N means the number of students included in each category.*

### 3.1 The impact of multilevel metacognition on group performance across different group categories

The significance of fostering group metacognition via multilevel interactions has been highlighted. This study investigates how multilevel metacognition influence group performance by examining its distribution and the differences observed across categories with differing achievement. Specifically, we analyzed the frequency and percentage of each encoding to identify metacognitive distribution characteristics and differences at the individual, interpersonal, and group levels of the different categories, as shown in Table 3. The distribution characteristics revealed that all three categories were characterized primarily by IPM. However, the H_T category exhibited the highest frequency of GM (Freq. = 116, Perc = 6.69%), the EF category had the most frequent IPM (Freq. = 1,108, Perc = 60.12%), and the L_T category displayed the highest frequency of IM (Freq. = 343, Perc = 22.11%). Specifically, H_T category showed the most frequent evaluations of individuals and interpersonal levels (IM_E: Freq. = 96, Perc = 26.45%; IPM_E: Freq. = 341, Perc = 32.95%), while EF category exhibited the most frequent monitoring of individuals and interpersonal levels (IM_M: Freq. = 195, Perc = 31.45%; IPM_M: Freq. = 576, Perc = 51.99%). From the perspective of GM development, certain categories show a higher tendency toward GM, which plays a more significant role in their performance, as demonstrated by their higher GM frequencies. The H_T category demonstrated the highest formation of GM, including group orientation, planning, monitoring, and evaluation. The EF category ranked second, especially in terms of group orientation. In contrast, the L_T category exhibited the lowest level of GM, especially in terms of group orientation and planning.

**Table 3:** Distribution of multilevel metacognition (total frequency of means within each category).

Category		Individual level	Interpersonal level	Group level	C	Ot
		IM (O, P, M, E)	IPM (O, P, M, E)	GM (O, P, M, E)		
H_T category (*N* = 20)	Freq.	363 (22, 106, 139, 96)	1,035 (36, 194, 464, 341)	116 (3, 14, 54, 45)	191	30
	Perc	20.92% (6.06, 29.20, 38.29, 26.45%)	59.65% (3.48, 18.74, 44.83, 32.95%)	6.69% (2.59, 12.07, 46.55, 38.79%)	11.01%	1.73%
EF category (*N* = 23)	Freq.	407 (15, 128, 195, 69)	1,108 (27, 217, 576, 288)	70 (0, 6, 32, 32)	148	110
	Perc	22.08% (3.69, 31.45, 47.91, 16.95%)	60.12% (2.44, 19.58, 51.99, 25.99%)	3.80% (0.00, 8.57, 45.71, 45.71%)	8.03%	5.97%
L_T category (*N* = 19)	Freq.	343 (11, 95, 153, 84)	731 (3, 150, 340, 238)	32 (0, 2, 17, 13)	216	229
	Perc	22.11% (3.21, 27.70, 44.61, 24.49%)	47.13% (0.41, 20.52, 46.51, 32.56%)	2.06% (0.00, 6.25, 53.13, 40.63%)	13.93%	14.76%

*N means the number of students included in each category.*

Second, a one-way ANOVA was conducted to examine the effects and variations in metacognitive levels on the performance of various categories. The differences in IM and IPM among the three categories did not significantly differ (IM: *F* = 0.038, *p* = 0.963; IPM: *F* = 0.637, *p* = 0.549), while the differences in GM were statistically significant (*F* = 5.6, *p* = 0.023 < 0.05). Additionally, the effect quantity (*η*^2^) was 0.542, indicating a moderate effect, as shown in Table 4. These findings suggest significant disparities in GM across collaborative achievement categories. The results confirm that while IM and IPM are present across categories, they do not significantly account for the variance in group performance. Instead, GM stands out as the key to differentiating high-performing categories (such as H_T category) from lower-performing one. The GM emerges as a crucial determinant of group performance, surpassing the influence of individuals and the IPM. Furthermore, fostering the development of GM has proven beneficial for enhancing group performance within small groups.

**Table 4:** One-way ANOVA results for GM in different categories.

Category	*N*	Mean	SD	SE	*F*	*p*	*η*^2^
H_T category	4	24.00	9.31	4.65	5.90*	0.02	0.54
EF category	5	14.00	4.18	1.87			
L_T category	4	8.50	5.45	2.72			

*N means the number of groups included in each category.*

### 3.2 Sequential characteristics and patterns of multilevel metacognitive interactions in categories with different achievements

**Figure 2:**
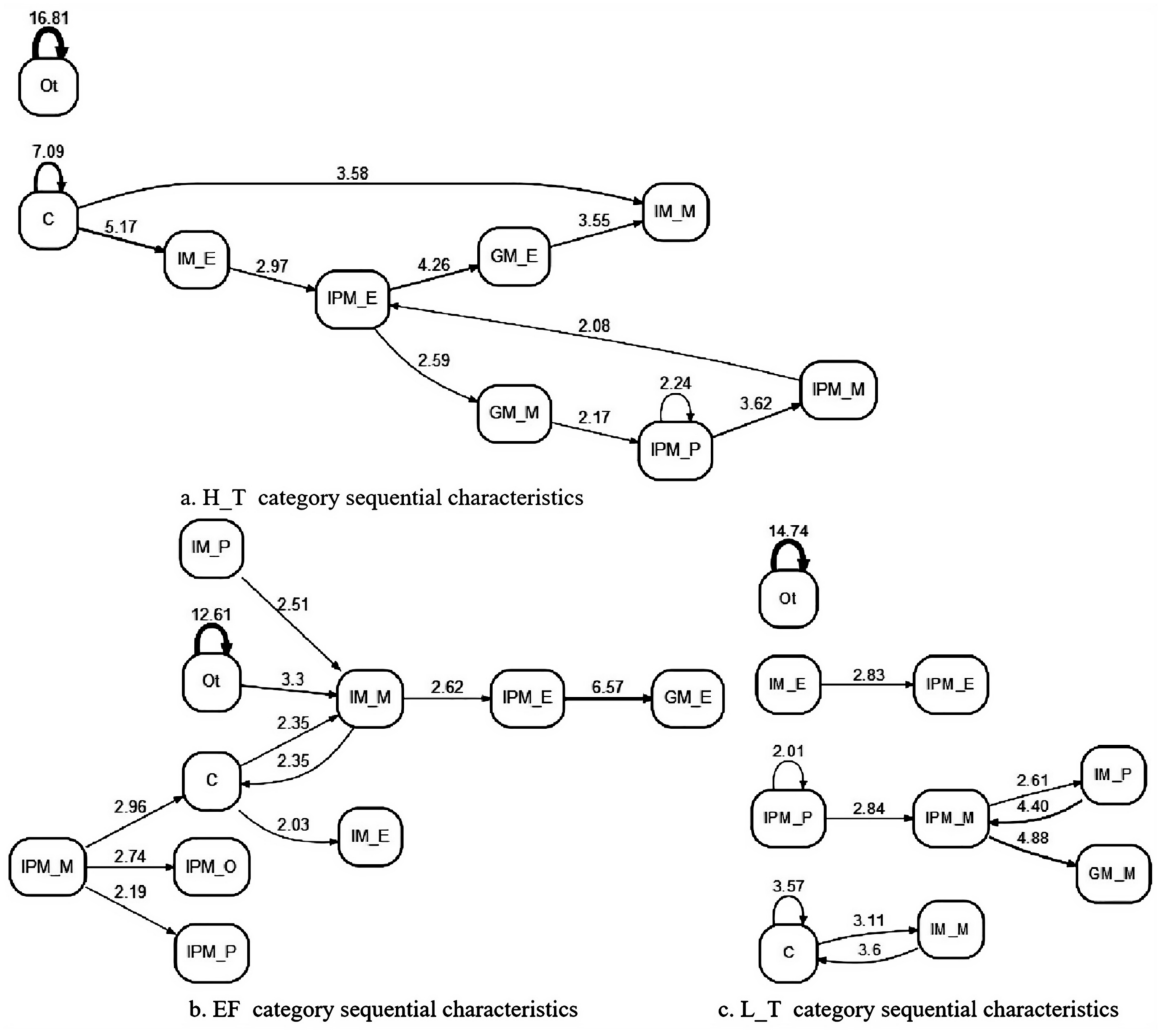
Group metacognitive sequential characteristics of different categories.

This study used LSA to analyze the sequential characteristics of multilevel metacognitive interactions. To ensure result reliability, transitions with Fre ≥ 12 and *z* > 1.96 were selected, as shown in Figure 2. Among them, the H_T category exhibited 12 direct transformations, 7 occurring between different levels and 5 occurring within a single level (see Figure 2a). EF category also demonstrated 12 direct transformations, with 9 occurring between different levels and 3 occurring within a single level (see Figure 2b). However, the L_T category generated 10 significant transitions, with 6 occurring between different levels and 4 occurring within a single level (see Figure 2c). All categories showed the highest conversion toward IPM, with GM mainly formed through the IPM. The H_T category displayed greater conversion toward IPM, while the L_T category exhibited a poor correlation between behaviors. Additionally, cognitive sharing primarily facilitates individual monitoring (C → IM_M), while other behavior is typically characterized by its ongoing nature (Ot → Ot).

At the individual level, the H_T category exhibited the transition from individual evaluation to interpersonal evaluation (IM_E → IPM_E), whereas the EF category exhibited the transitions from individual planning to individual monitoring or cognitive sharing, and from individual monitoring to interpersonal evaluation (IM_P → IM_M, IM_M → C and IM_M → IPM_E). In addition, the L_T category exhibited the transitions from individual planning to interpersonal evaluation (IM_E → IPM_E) and from individual evaluation to interpersonal monitoring (IM_P → IPM_M). At the interpersonal level, the H_T category exhibited the following transitions from interpersonal evaluation to group monitoring and evaluation (IPM_E → GM_M, IPM_E → GM_E) while forming the transition from internal planning, monitoring, and evaluation sequences at the interpersonal level (IPM_P → IPM_M, IPM_M → IPM_E). The EF category supports interpersonal planning and goal setting through interpersonal monitoring (IPM_M → C, IPM_M → IPM_P, IPM_M → IPM_O), while reaching agreement through interpersonal evaluation and group evaluation (IPM_E → GM_E). The L_T category exhibited the following transitions from interpersonal planning to interpersonal monitoring and further to group monitoring (IPM_P → IPM_M, IPM_M → GM_M), while interpersonal monitoring also supports individual planning (IPM_M → IM_P). At the group level, the H_T category demonstrated the transition from group evaluation to individual monitoring (GM_E → IM_M) and the transition from group evaluation to interpersonal planning (GM_E → IPM_P), whereas the other categories lacked transitions from GM to other levels. Sequence analysis revealed that transformations occurred mainly between different levels of metacognition, focusing on initiating or directing toward IPM. IM plays a pivotal role in enhancing metacognitive interaction effectiveness. Additionally, transformations originating from GM represent a significant process influencing group transactivity.

**Figure 3:**
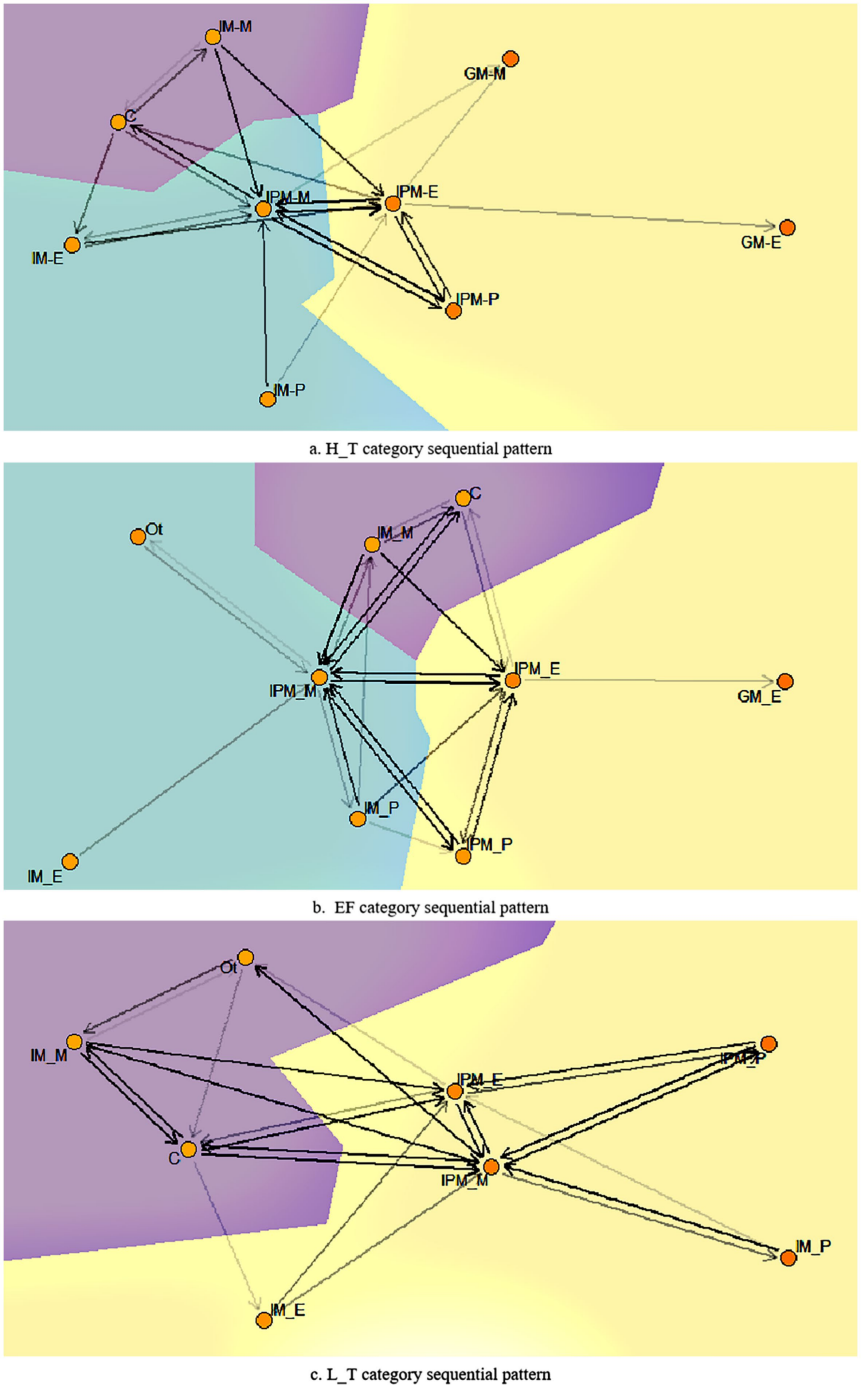
Group metacognitive patterns of different categories.

This study used cSPADE to mine group metacognitive patterns, as shown in Figure 3, to analyze how learners form GM and explain differences across categories. Each category comprises four or five groups, enabling the identification of commonalities and the establishment of a coherent pattern. The circles in the pattern represent specific events involving learners in the collaborative process. Edge weights within the pattern were determined by transition frequency, and symmetry and local aggregation were calculated based on edge weights to illustrate the optimal cluster structure of micro processes. The background color is used to distinguish between different trends, and the background area reflects the proportion of the trend in the overall pattern. In Figure 3, all three categories develop a group metacognitive pattern centered on interpersonal monitoring and evaluation, including three important behavioral trends: bidirectional transformation between cognitive sharing and individual monitoring, extension of IM to IPM, and a trend toward GM influenced by IPM. H_T category exhibited prominent transitions between IM and IPM, purposefully extending to GM. Individuals in this category actively took responsibility for their own metacognitive processes but also extended this responsibility to the group level. This sustained effort resulted in the emergence of group monitoring and group evaluation (see Figure 3a). EF category spends effort coordinating group activities through interpersonal monitoring. Group members focus on adjusting and achieving each other’s goals, and IM plays a prominent role, particularly in monitoring and planning. This focus on IM leads to strong interactions within and between IM and IPM, ultimately resulting in GM, such as group evaluation (see Figure 3b). Conversely, L_T category dedicates more effort to coordination, with relatively limited efforts in IM, resulting in a low interaction mode guided by IM and IPM without extending to group-level dynamics (see Figure 3c). This finding is consistent with research findings on metacognitive similarity as a marker for identifying group metacognition (Robinson and Gonnerman, 2020). And in contrast to inter-level correlations (Zheng et al., 2023), our findings add new understanding of how metacognitive processes at each level transition and interact across levels in collaborative learning.

To identify key transformations in group patterns and understand the rules contributing to metacognitive processes, the study analyzed the frequent sequence rules of different categories, as shown in Table 5. The support level in the table represents the frequency of rules, while the confidence level represents the probability of rules being true. We set the minimum support for frequent rule occurrences to 0.25 and the minimum confidence level to 0.6. All three categories have established rules ranging from individual monitoring to interpersonal monitoring, indicating that individual monitoring triggers interpersonal monitoring. Additionally, interpersonal monitoring is central to group metacognitive patterns, and supporting individual monitoring is vital for activating group metacognitive process. Moreover, all rules are guided by interpersonal monitoring, which plays a crucial role in forming GM. The H_T category formed the most rules, followed by the EF category, while the L_T category had the fewest rules. The interpersonal monitoring rules of the H_T category and EF category are triggered by continuous interpersonal monitoring and planning, suggesting that interpersonal planning may play an important role in forming GM.

**Table 5:** Group metacognitive frequent sequence rules for collaborative categories.

Category	Rules	Support	Confidence
H_T category	{IPM-M}>	0.43	0.62
	{IPM-M}>	0.30	0.72
	{IPM-M}>	0.30	0.68
	{IPM-E}>	0.26	0.63
EF category	{IPM_M}>	0.51	0.64
	{IPM_M}>	0.39	0.77
	{IPM_M}>	0.32	0.66
L_T category	{IPM_M}>	0.32	0.75
	{IPM_M}>	0.30	0.66

## 4 Discussion and implications

### 4.1 Interpersonal metacognition is the main theme, but group metacognition is key to influencing collaborative learning

The descriptive analysis supported the findings from previous research. The overall impact of varying levels of metacognition on group regulation and performance across all categories was examined. IPM, particularly interpersonal monitoring, prevails in metacognitive interaction, validating the idea that sharing metacognitive thinking enhances opportunities for group members to engage in metacognitive interactions (Haataja et al., 2022a,b; Iiskala et al., 2021). Furthermore, the greater frequency of interpersonal evaluations aligns with the need for ongoing coordination and calibration among members through evaluations to maintain consistent group understanding during metacognitive interactions (Kwon et al., 2014). Moreover, consistent with the findings of other studies (Järvelä et al., 2021; Stehle, 2022), individual monitoring emerged as the most common individual metacognitive process, indicating that learners activate other levels of metacognition through autonomous monitoring. Additionally, group members are more likely to establish group consensuses through continuous monitoring and evaluation. The emergence of metacognitive similarity as a hallmark of GM (Robinson and Gonnerman, 2020) underscores the potential significance of monitoring and evaluation. While group goals and plans were less frequent, significant disparities were observed between group categories that did and did not engage in these aspects (Hogenkamp et al., 2021). This underscores the potential importance of group goals and plans in shaping the GM, warranting further exploration.

Specifically, significant differences in GM were observed among group categories with varying achievements, indicating GM is crucial to differences in collaborative learning performance. Unlike previous studies that directly examined the correlation between GM and collaborative learning achievement (Liu et al., 2023), this study identified group categories based on differences in individual preparation and group performance. It then analyzed variations in GM under different achievement changes to assess the impact of GM on collaborative learning. Consistent with Liu et al.’s research (Liu et al., 2023), this study also confirmed that GM facilitates learners’ gains from collaborative learning. However, the extent to which learners benefit from collaborative learning is linked to the degree of GM development. Specifically, a higher level of GM corresponds to learners receiving benefits from collaborative learning more easily, as observed in the H_T category in this study. This combined assessment of individual preparation and group task performances focuses on the influence of individuals on the group. GM supports explaining how individual preparation performance relates to changes in group task performance. Additionally, this study aligns with Smith’s research (Smith and Mancy, 2018), further validating that more successful individuals exhibit more metacognitive processes, such as the EF category. Individual preparation performance helps explain the role and actual effects of IM within groups, contributing to our understanding of the in-depth effects of multilevel metacognition on group performance. Multilevel process analysis is necessary to correspond with the hierarchical composition and changes in group performance.

### 4.2 Differences in individual and interpersonal metacognition support group metacognition in different categories

Based on the sequential interaction of the GM with different levels of metacognition, it has been confirmed that the IM is the foundation of other levels of metacognition (Socratous and Ioannou, 2022). Metacognitive sharing promotes common understanding (Gandolfi et al., 2023), and whether IM promotes metacognitive co-construction is crucial for influencing GM. For instance, at the individual level, H_T category and L_T category interact through metacognitive sharing from individual evaluation to interpersonal evaluation and individual planning to interpersonal monitoring. In contrast, EF category focuses on metacognitive co-construction triggered by self-directed monitoring of individual plans and the co-construction of individual monitoring to control cognitive activities and interpersonal evaluation. The IPM plays a crucial role in shaping the IM and forming the GM. Variations in the process of IPM are a significant factor contributing to different group patterns. For instance, at the interpersonal level, H_T category experiences a transition from planning to monitoring, then to evaluation, and finally to group monitoring or evaluation, thereby establishing a path for GM formation driven by IPM. In contrast, L_T category engages in sharing plans and co-constructing monitoring to group monitoring and individual plans, suggesting that high-performance categories are more inclined to extend interpersonal metacognition to the group level, while low-performance category need to exert effort to adjust IM. Whether GM contributes to differences in collaborative learning performance is a crucial consideration. Only the H_T category experienced a significant shift in GM, impacting IM and IPM, suggesting that GM further promotes metacognition engagement. However, achieving this influence may require external support.

Research indicates that all three group categories develop a metacognitive interaction pattern centered on IPM_M and IPM_E. However, only H_T category and EF category exhibited GM in this pattern. The lack of frequent rules related to the GM may contribute to a lower GM. Specifically, the sequential pattern yields additional insights into how IM and IPM contribute to GM. First, cognitive sharing and IM lay the groundwork for subsequent shifts in trends. While cognitive sharing and individual monitoring create opportunities for metacognitive interactions (Haataja et al., 2022a,b), they come with the caveat of reducing the effort required for social coordination. For instance, within the H_T category and EF category, cognitive sharing and self-monitoring promote participation in metacognitive interactions. In contrast, despite a similar trend in the L_T category, social coordination is needed, and social conflict may exist. Moreover, IM makes additional contributions by helping learners benefit from interpersonal interactions, thus reducing the effort required for GM (Gandolfi et al., 2023). For instance, compared with other categories, EF category generates more IPM through individual planning and evaluation. Second, interpersonal monitoring is the center of the second trend of all categories and is the key to shifting behavioral trends, signifying that group members have the opportunity to develop partial shared understanding through metacognitive co-construction. Finally, high-performing categories support more interpersonal metacognitive co-construction and are more likely to extend IPM to GM. In H_T category, interpersonal monitoring and evaluation consistently lead to group monitoring or evaluation, suggesting more negotiation processes within such groups (Gandolfi et al., 2023). This underscores the positive interdependence, increased metacognitive interactions, and greater team productivity of active metacognitive categories compared with the passive one. However, the pattern lacks representation of the sequence characterization of GM on individuals and IPM, requiring further validation.

### 4.3 Comprehensive understanding of the development of group metacognition

The statistical analysis, sequential characteristics, and patterns of metacognitive interaction suggest that each collaborative group member’s participation depends on coordinated metacognitive knowledge, monitoring, and control. Learners’ potential metacognitive abilities are realized through group collaboration (Badhe et al., 2023), and their learning outcomes are influenced by GM. Failure to calibrate metacognitive monitoring or control may endanger collaboration and its outcomes (Winne et al., 2013). This can also further explain why interpersonal metacognition among H_T category can activate IM. H_T category learners benefit from IPM by calibrating each other’s metacognition and promoting their metacognitive contributions (Kolić-Vehovec et al., 2022), leading to the construction of GM (Molenaar et al., 2014). In EF category, accurate IM enables effective IPM, promoting the sharing and co-construction of metacognition at the interpersonal level by identifying consistency between metacognitions (Gandolfi et al., 2023) and forming GM. Conversely, in the L_T category, less individual metacognitive participation and increased interpersonal metacognition and coordination did not positively impact the group level metacognition. This may result in poor individual metacognitive accuracy (Haataja et al., 2022a), with the IPM failing to calibrate or activate IM. Additionally, interpersonal metacognitive support may be ignored, resulting in less GM and hindering effective collaboration (Järvelä et al., 2021). This finding underscores the important role of individual metacognitive effort and interpersonal metacognitive alignment in forming GM. Individual metacognitive effort helps establish effective metacognitive interaction, serving as the foundation of GM. Interpersonal metacognitive alignment combines IM and group cognition (Gandolfi et al., 2023), promotes the contribution of IM by group members, and effectively links IM and GM, forming a virtuous cycle. The contribution of IM or IPM to the collaborative group, as well as the impact of GM, may influence what learners gain from collaborative learning (Paans et al., 2019). H_T category benefits individuals and group through metacognitive interactions, while EF category maximizes the impact of metacognitive efforts on individuals through such interactions. In contrast, L_T category either ignore or fail to support sustained metacognitive interactions, leading to improved performance after completing collaborative tasks. This further confirms that GM affects collaborative learning performance.

### 4.4 Limitations and future research

This research has several limitations. First, the small sample size limits the generalizability and statistical power of the results for detecting group metacognitive pattern effects. Additionally, the data were collected from a large, selective public university and may not represent all college students. Future research should employ a larger sample size and conduct more detailed investigations into how different collaborative learning groups form and develop GM. Second, while the study grouped learners based on their metacognitive levels, it did not analyze their prior knowledge or learning motivation. Due to the mid-academic year of data collection, future research should explore how learners’ individual characteristics affect GM. Third, this study focuses on how different levels of metacognition within a group interact to facilitate GM development. Although the impacts of individual and IPM on GM density were identified, future research could further explore the significance and predictive power of the impact of IM and IPM on GM. This could enable the development of personalized interventions and support. Concurrently, a deeper analysis of individual learner characteristics within the overall group and different group categories is warranted. Additionally, further exploration of how GM translates to IM or IPM is needed. Finally, this study was conducted in a natural collaborative learning environment, and the impact of support tools on GM was not considered. Hence, further exploration is needed to determine whether different support tools affect the GM.

## 5 Conclusion

The multilevel feature of metacognition significantly influences GM. However, traditional analysis methods often fail to connect multiple levels, limiting insights into how complex collaborative metacognitive interaction processes shape GM. From a methodological perspective, a combined approach using sequence analysis and process mining was used to examine the multilevel features of collaborative metacognitive interactions. The research results preliminarily validate the effectiveness of the coding framework in conceptualizing multilevel metacognition, as well as the significant impact of GM on collaborative learning performance. They emphasize the importance of IPM for GM and the critical role of monitoring and evaluating transitions across the individual, interpersonal, and group levels. IPM plays an important mediating role in promoting students’ metacognitive interaction while using GM. Moreover, it is essential to explore how to support IM more effectively and to understand how individual characteristics may impact on low IM interactions. The positive impact of GM on metacognitive interactions necessitates specific support to optimize these interactions. Additionally, the role of social behavior on group metacognition also deserves further exploration. The group metacognitive pattern can guide educational practice and provide guidance for researchers in the transition of L_T category to H_T and EF categories, as well as provide a basis for supporting researchers in creating H_T category or EF category metacognitive patterns. Moreover, the fusion analysis method proposed in this study mitigates the impact of ontology flatness and method assumptions, enabling researchers to conduct more comprehensive temporal analysis.

## Data Availability

The original contributions presented in the study are included in the article/supplementary material, further inquiries can be directed to the corresponding authors.

## References

[ref1] Badhe V. Dasgupta C. Rajendran R. (2023). Investigating teams’ socially shared metacognitive regulation (SSMR) and transactivity in project-based computer supported collaborative learning environment. Proceedings of the 16th international conference on educational data mining. Bengaluru, India: International Educational Data Mining Society.

[ref2] Bakhtiar A. Hadwin A. F. (2020). Dynamic interplay between modes of regulation during motivationally challenging episodes in collaboration. Frontline Learn. Res. 8, 1–34. doi: 10.14786/flr.v8i2.561

[ref3] Biasutti M. Frate S. (2018). Group metacognition in online collaborative learning: validity and reliability of the group metacognition scale (GMS). Educ. Technol. Res. Dev. 66, 1321–1338. doi: 10.1007/s11423-018-9583-0

[ref4] Briñol P. DeMarree K. (2012). Social metacognition. New York: Psychology Press.

[ref5] Chalmers C. (2009). Primary students’ group metacognitive processes in a computer supported collaborative learning environment (Doctoral dissertation): Queensland University of Technology.

[ref6] De Backer L. Van Keer H. Valcke M. (2022). The functions of shared metacognitive regulation and their differential relation with collaborative learners’ understanding of the learning content. Learn. Instr. 77:101527. doi: 10.1016/j.learninstruc.2021.101527

[ref8] Fleiss J. (1981). Statistical itletlzods for rates and proportions. New York: John Wiley and Sons.

[ref9] Gandolfi G. Pickering M. J. Garrod S. (2023). Mechanisms of alignment: shared control, social cognition and metacognition. Philos. Trans. R. Soc. B 378:20210362. doi: 10.1098/rstb.2021.0362, PMID: 36571124 PMC9791477

[ref10] Haataja E. Dindar M. Malmberg J. Järvelä S. (2022a). Individuals in a group: metacognitive and regulatory predictors of learning achievement in collaborative learning. Learn. Individ. Differ. 96:102146. doi: 10.1016/j.lindif.2022.102146

[ref11] Haataja E. Malmberg J. Dindar M. Järvelä S. (2022b). The pivotal role of monitoring for collaborative problem solving seen in interaction, performance, and interpersonal physiology. Metacogn. Learn. 17, 241–268. doi: 10.1007/s11409-021-09279-3

[ref12] Halmo S. M. Bremers E. K. Fuller S. Stanton J. D. (2022). “Oh, that makes sense”: social metacognition in small-group problem solving. CBE—life sciences. Education 21:ar58. doi: 10.1187/cbe.22-01-0009, PMID: 35998162 PMC9582819

[ref13] Hogenkamp L. van Dijk A. M. Eysink T. H. (2021). Analyzing socially shared regulation of learning during cooperative learning and the role of equal contribution: a grounded theory approach. Educ. Sci. 11:512. doi: 10.3390/educsci11090512

[ref14] Huang Y. Zheng X. Kim H. (2021). Effects of scaffolding types and individual metacognition levels on learning achievement in online collaborative argumentation. Educ. Technol. Int. 22, 311–339. doi: 10.23095/ETI.2021.22.2.311

[ref15] Hurme T.-R. Järvelä S. Merenluoto K. Salonen P. (2015). “What makes metacognition as socially shared in mathematical problem solving?” in Metacognition: fundaments, applications, and trends: a profile of the current state-of-the-art. ed. Peña-Ayala A. (New York: Springer), 259–276.

[ref16] Hurme T.-R. Merenluoto K. Järvelä S. (2009). Socially shared metacognition of pre-service primary teachers in a computer-supported mathematics course and their feelings of task difficulty: a case study. Educ. Res. Eval. 15, 503–524. doi: 10.1080/13803610903444659

[ref17] Iiskala T. Vauras M. Lehtinen E. Salonen P. (2011). Socially shared metacognition of dyads of pupils in collaborative mathematical problem-solving processes. Learn. Instr. 21, 379–393. doi: 10.1016/j.learninstruc.2010.05.002

[ref18] Iiskala T. Volet S. Jones C. Koretsky M. Vauras M. (2021). Significance of forms and foci of metacognitive regulation in collaborative science learning of less and more successful outcome groups in diverse contexts. Instr. Sci. 49, 687–718. doi: 10.1007/s11251-021-09558-1

[ref19] Järvelä S. Malmberg J. Sobocinski M. Kirschner P. A. (2021). “Metacognition in collaborative learning” in International handbook of computer-supported collaborative learning. eds. Cress U. Rosé C. Wise A. F. Oshima J. (New York: Springer), 281–294.

[ref20] Kelly D. (2018). The individual and social complexities of metacognition in education-based learning (Doctoral dissertation): George Mason University.

[ref21] Kim Y. R. Moore T. J. (2019). Multiple levels of metacognition: circumstances interfering with students' spontaneous metacognitive activities. J. Educ. Res. Pract. 9, 158–178. doi: 10.5590/JERAP.2019.09.1.12

[ref22] Kolić-Vehovec S. Pahljina-Reinić R. Rončević Zubković B. (2022). Effects of collaboration and informing students about overconfidence on metacognitive judgment in conceptual learning. Metacogn. Learn. 17, 87–116. doi: 10.1007/s11409-021-09275-7

[ref23] Kwon K. Hong R.-Y. Laffey J. M. (2013). The educational impact of metacognitive group coordination in computer-supported collaborative learning. Comput. Hum. Behav. 29, 1271–1281. doi: 10.1016/j.chb.2013.01.003

[ref24] Kwon K. Liu Y.-H. Johnson L. P. (2014). Group regulation and social-emotional interactions observed in computer supported collaborative learning: comparison between good vs. poor collaborators. Comput. Educ. 78, 185–200. doi: 10.1016/j.compedu.2014.06.004

[ref25] Li W. Liu C. Y. Tseng J. C. (2023). Development of a metacognitive regulation-based collaborative programming system and its effects on students' learning achievements, computational thinking tendency and group metacognition. Br. J. Educ. Technol. 55, 318–339. doi: 10.1111/bjet.13358

[ref27] Liu C. Y. Li W. Huang J. Y. Lei L. Y. Zhang P. R. (2023). Collaborative programming based on social shared regulation: an approach to improving students' programming achievements and group metacognition. J. Comput. Assist. Learn. 39, 1714–1731. doi: 10.1111/jcal.12828

[ref28] Meijer J. Veenman M. V. van Hout-Wolters B. H. (2006). Metacognitive activities in text-studying and problem-solving: development of a taxonomy. Educ. Res. Eval. 12, 209–237. doi: 10.1080/13803610500479991

[ref29] Molenaar I. Chiu M. M. Sleegers P. van Boxtel C. (2011). Scaffolding of small groups’ metacognitive activities with an avatar. Int. J. Comput.-Support. Collab. Learn. 6, 601–624. doi: 10.1007/s11412-011-9130-z, PMID: 27069424 PMC4811593

[ref30] Molenaar I. Sleegers P. van Boxtel C. (2014). Metacognitive scaffolding during collaborative learning: a promising combination. Metacogn. Learn. 9, 309–332. doi: 10.1007/s11409-014-9118-y

[ref1003] Nelsons T. O. (1999). Cognition versus metacognition. In Sternberg R. J. editor. The nature of cognition, Cambridge, MA: MIT Press. 625–641.

[ref32] Olesova L. Liao D. Rytikova I. Boicu M. Foxwell H. J. (2023). Metacognition in graduate engineering courses. Paper presented at the 2023 ASEE annual conference & exposition. Baltimore, Maryland.

[ref33] Paans C. Onan E. Molenaar I. Verhoeven L. Segers E. (2019). How social challenges affect children’s regulation and assignment quality in hypermedia: a process mining study. Metacogn. Learn. 14, 189–213. doi: 10.1007/s11409-019-09204-9

[ref1002] Rapchak M. E. (2018). Collaborative learning in an information literacy course: The impact of online versus face-to-face instruction on social metacognitive awareness. J. Acade. Libraria. 44, 383–390.

[ref34] Robinson B. Gonnerman C. (2020). “Enhancing cross-disciplinary science through philosophical dialogue: evidence of improved group metacognition for effective collaboration”, in The toolbox dialogue initiative. eds. Hubbs G. O’rourke M. Orzack S. H. (Boca Raton: CRC Press), 127–141.

[ref35] Saqr M. López-Pernas S. (2023). The temporal dynamics of online problem-based learning: why and when sequence matters. Int. J. Comput.-Support. Collab. Learn. 18, 11–37. doi: 10.1007/s11412-023-09385-1

[ref36] Siegel M. A. (2012). Filling in the distance between us: group metacognition during problem solving in a secondary education course. J. Sci. Educ. Technol. 21, 325–341. doi: 10.1007/s10956-011-9326-z

[ref37] Smith J. M. Mancy R. (2018). Exploring the relationship between metacognitive and collaborative talk during group mathematical problem-solving–what do we mean by collaborative metacognition? Res. Math. Educ. 20, 14–36. doi: 10.1080/14794802.2017.1410215

[ref38] Socratous C. Ioannou A. (2019). “An empirical study of educational robotics as tools for group metacognition and collaborative knowledge construction” in A wide lens: combining embodied, enactive, extended, and embedded learning in collaborative settings, 13th international conference on computer supported collaborative learning (CSCL) 2019. eds. Lund K. Niccolai G. P. Lavoué E. Hmelo-Silver C. Gweon G. Baker M., vol. 1 (Lyon, France: International Society of the Learning Sciences), 192–199.

[ref39] Socratous C. Ioannou A. (2022). Evaluating the impact of the curriculum structure on group metacognition during collaborative problem-solving using educational robotics. TechTrends 66, 771–783. doi: 10.1007/s11528-022-00738-5

[ref40] Stanton J. D. Sebesta A. J. Dunlosky J. (2021). Fostering metacognition to support student learning and performance. CBE Life Sc. Educ. 20:fe3. doi: 10.1187/cbe.20-12-0289 33797282 PMC8734377

[ref41] Stehle S. M. (2022). “Does that make sense?” A mixed methods study investigating high school physics students’ use of metacognition while solving physics problems (Doctoral dissertation): George Mason University.

[ref42] Su Y. Li Y. Hu H. Rosé C. P. (2018). Exploring college English language learners’ self and social regulation of learning during wiki-supported collaborative reading activities. Int. J. Comput.-Support. Collab. Learn. 13, 35–60. doi: 10.1007/s11412-018-9269-y

[ref43] Tang H. Arslan O. Xing W. Kamali-Arslantas T. (2022). Exploring collaborative problem solving in virtual laboratories: a perspective of socially shared metacognition. J. Comput. High. Educ. 35, 296–319. doi: 10.1007/s12528-022-09318-1

[ref44] Teng F. (2020). Tertiary-level students’ English writing performance and metacognitive awareness: a group metacognitive support perspective. Scand. J. Educ. Res. 64, 551–568. doi: 10.1080/00313831.2019.1595712

[ref45] Thompson L. Cohen T. R. (2012). “Metacognition in teams and organizations”, in Soc. Metacogn. eds. Brin˜ol P. DeMarree K. G. (New York: Psychology Press), 283–302.

[ref46] Uslu N. A. Durak H. Y. (2022). Predicting learner autonomy in collaborative learning: the role of group metacognition and motivational regulation strategies. Learn. Motiv. 78:101804. doi: 10.1016/j.lmot.2022.101804

[ref48] Waller M. J. Uitdewilligen S. Rico R. Thommes M. S. (2021). “Interaction pattern and trajectory analysis for studying group communication”, in The emerald handbook of group and team communication research. eds. Beck S. J. Keyton J. Poole M. S. (Bingley: Emerald Publishing Limited), 135–153.

[ref49] Winne P. H. Hadwin A. F. Perry N. E. (2013). “Metacognition and computer-supported collaborative learning”, in The international handbook of collaborative learning. eds. Hmelo-Silver C. O’Donnell A. Chan C. Chinn C. (New York: Taylor & Francis), 462–479.

[ref51] Zheng X. L. Huang J. Xia X. H. Hwang G. J. Tu Y. F. Huang Y. P.. (2023). Effects of online whiteboard-based collaborative argumentation scaffolds on group-level cognitive regulations, written argument skills and regulation patterns. Comput. Educ. 207:104920. doi: 10.1016/j.compedu.2023.104920

[ref52] Zheng L. Li X. Zhang X. Sun W. (2019). The effects of group metacognitive scaffolding on group metacognitive behaviors, group performance, and cognitive load in computer-supported collaborative learning. Internet High. Educ. 42, 13–24. doi: 10.1016/j.iheduc.2019.03.002

